# Optimization of chemical induction for evaluation of endogenous retroviruses in different species

**DOI:** 10.1186/1742-4690-8-S2-P38

**Published:** 2011-10-03

**Authors:** Hailun Ma, Yunkun Ma, Wenbin Ma, Dhanya K Williams, Teresa A Galvin, Syed Shaheduzzaman, Arifa S Khan

**Affiliations:** 1 Laboratory of Retroviruses, Division of Viral Products, Center for Biologics Evaluation and Research, U.S, Food and Drug Administration, Bethesda, Maryland, 20892, USA

## Background

Chemical inducers such as 5-azacytidine (AzaC) and 5'-iodo-2'deoxyuridine (lUdR) have been used to discover and characterize endogenous retroviruses from rodent and avian speciecs, for example KBALB mouse cells. We found that induction conditions that have been optimized for mouse cells were not successful in inducing retrovirus from cell lines of other species, including nonhuman primates. Therefore, we developed a step-wise strategy based upon identification of critical parameters for endogenous retrovirus induction in mouse cells for optimizing induction conditions for non-murine cells. Using this approach, we have determined optimum conditions for investigating inducible endogenous retroviruses from Vero cells, which are of African green monkey (AGM) origin, a species that has never been reported to produce endogenous retroviruses [1].

## Materials and methods

Based upon a step-wise induction strategy [2], Vero cell growth characteristics such as growth curve, population doubling time, and cell cycle phase were determined; drug dose range was obtained by evaluating cell toxicity and cell recovery using different drug concentrations (lUdR, 50 - 3,200 ug/ml; AzaC, 0.2125 - 40 ug/ml; and NaBut, 1 - 6 mM). Cellular RNAs were tested for endogenous retrovirus activation using virus-specific PCR assays and filtered supernatants were analyzed for virus particle production using a highly-sensitive RT assay and PCR assays [1]. Infectivity studies were done using various cell lines from different species based upon their susceptibility to known retrovirus infections. Cellular RNAs from untreated and AzaC-treated cells will be evaluated for virus induction using different emerging virus detection technologies.

## Results

The results demonstrated that endogenous retrovirus particles could be induced from Vero cells under optimized cell growth and drug treatment conditions. Molecular analysis indicated that the particles contained *gag*, *pol*, and *env* regions related to endogenous SERV and BaEV sequences previously reported in the AGM DNA. Biological studies showed no evidence of replication-competent particles using several target cell lines. Similarly, an RT activity could be induced from a dog cell line, which is another species with no evidence of retrovirus isolation. This is currently under investigation for further characterization.

## Conclusions

The induction of retrovirus particles from Vero cells was low and detected in cell-free supernatant only by using a highly sensitive PCR-based RT assay and virus-specific PCR assays. Further investigations of various emerging virus detection technologies used for novel virus discovery has demonstrated that induced viral RNAs could be detected in drug-treated cells using high throughput 454-massively parallel or deep sequencing: investigations with virus microarrays and long range PCR with mass spectrometry are ongoing. The results support that the combination of chemical induction strategy with sensitive broad virus detection technologies may be used for the discovery of novel endogenous retroviruses.
